# Trigonal-Sparing vs. Trigonal-Involved OnabotulinumtoxinA Injection for the Treatment of Overactive Bladder: A Systematic Review and Meta-Analysis

**DOI:** 10.3389/fneur.2021.651635

**Published:** 2021-10-08

**Authors:** Yuanshan Cui, Tong Cai, Tiantian Dong, Xiaoyi Zhang, Zhongbao Zhou, Youyi Lu, Yong Zhang, Jitao Wu, Zhenli Gao, Yongqiang Wang, Liying Dong

**Affiliations:** ^1^Department of Urology, The Affiliated Yantai Yuhuangding Hospital of Qingdao University, Yantai, China; ^2^Department of Urology, Beijing Tian Tan Hospital, Capital Medical University, Beijing, China; ^3^Yangzhou University, Yangzhou, China; ^4^People's Liberation Army of China Rocket Force Characteristic Medical Center, Department of Urology, Beijing, China

**Keywords:** onabotulinumtoxinA (BoNTA), overactive bladder (OAB), neurogenic detrusor overactivity (NDO), idiopathic detrusor overactivity, randomized controlled trials (RCT), meta-analysis

## Abstract

**Objective:** Overactive bladder (OAB) is a disease characterized by the presence of urinary urgency. We carried out a meta-analysis to assess the effectiveness and safety of trigonal-involved injection of onabotulinumtoxinA (BoNT-A) in comparison with the trigonal-sparing technique in cases with OAB [neurogenic detrusor overactivity (NDO) and idiopathic detrusor overactivity (IDO)].

**Methods:** Randomized controlled trials (RCTs) of BoNT-A injection for OAB were searched systematically by using EMBASE, MEDLINE, and the Cochrane Controlled Trials Register. The datum was calculated by RevMan version 5.3.0. The original references of relating articles were also reviewed.

**Results:** In total, six RCTs involving 437 patients were included in our analysis. For OAB, the trigone-including group showed a different patient symptom score (*p* = 0.03), complete dryness rate (*p* = 0.002), frequency of incontinence episodes (*p* = 0.01), detrusor pressure at maximum flow rate (*p* = 0.01), and volume at the first desire to void (*p* = 0.0004) compared with the trigone-sparing group. Also, a trigone-including intradetrusor injection demonstrated a significant improvement in the patient symptom score (*p* = 0.0004), complete dryness rate (*p* = 0.0002), frequency of incontinence episodes (*p* = 0.0003), detrusor pressure at maximum flow rate (*p* = 0.01), and volume at the first desire to void (*p* = 0.00006) compared with the trigone-sparing group for treatment of NDO. The adverse events rates were similar in both groups.

**Conclusions:** The meta-analysis has demonstrated that trigone-including BoNT-A injection was more effective compared with the trigone-sparing injection for the treatment of OAB, especially for NDO.

## Introduction

According to the International Continence Society (ICS), overactive bladder (OAB) is a disease characterized by the presence of urinary urgency, usually accompanied by frequency and nocturia, with or without urgency urinary incontinence (UUI), in the absence of urinary tract infection (UTI) or other clear pathology (1). OAB is a common disease that threatens the quality of human life severely. The symptoms of OAB were disturbing chronic disease and imposed an economic burden in many patients (2). The incidence of OAB increased significantly with age from 11% up to 20% between the ages of 40 and 60 years for both men and women (3, 4). OAB was considered as a symptom complex, and it includes idiopathic detrusor overactivity (IDO) or neurogenic detrusor overactivity (NDO). According to the ICS, IDO was defined as the occurrence of involuntary detrusor contractions during filling cystometry without infected and pathological changes. NDO was detrusor overactivity (DO) caused by various neurologic diseases such as brain tumors, dementia, and spinal cord injury (SCI) (5). Although the causes of NDO and IDO are different, they show many similar symptoms and treatment methods.

For treating this disease, there are many methods, including bladder and behavioral training, drug treatment, onabotulinumtoxinA (BoNT-A), neuromodulation, and surgical therapies. Bladder and behavioral training was the first-line treatment for OAB, and drug treatment, such as the use of anticholinergics, was the second-line treatment; low compliance can affect the treatment result in the mentioned two treatments (3). In these circumstances, onabotulinumtoxinA has been advised as an alternative treatment for patients.

BoNT-A is a poisonous potent neurotoxin produced by the anaerobic bacterium clostridium botulinum and related species (6). According to antigenic and serological classification, there are eight distinguishable exotoxins of BoNT (A, B, C_1_, C_2_, D, E, F, and G), and type A had a better effect in durative time (7). BoNT-A inhibits muscle contractions on muscle by binding strongly to presynaptic cholinergic nerve terminals at the neuromuscular junction to inhibit acetylcholine release.

According to the American Urological Association and European Association of Urology guidelines recommendations, BoNT-A has been recommended as an alternative treatment (8, 9). Intravesical BoNT-A injections have achieved the satisfactory clinical effect; it could increase bladder capacity, decreased sense of urgency, and improved other symptoms in patients with NDO or IDO (10–13). In the past 10 years, the drugs were injected into different sites of the bladder wall with a cystoscope. The most common adverse reactions following the administration of the toxin reported in the clinical research were urinary retention and urinary tract infection (14). The trigone of the bladder has a large number of sensory nerve fibers (15), which might improve effectiveness with trigonal injections. Recent research also showed that trigonal BoNT-A injection had a less adverse effect and shorter treatment time compared to that of bladder body injections (16).

BoNT-A injection is an effective alternative treatment. Unfortunately, there is no standardized way of injecting BoNT-A for the treatment of OAB. Therefore, a systematic review and a meta-analysis of randomized controlled trials (RCTs) were performed to assess the effectiveness and safety of trigonal-involved injection of BoNT-A comparison with the trigonal-sparing technique in cases with OAB (NDO and IDO).

## Materials and Methods

### Study Design

A systematic review of RCTs was carried out using the preferred reporting items for the meta-analyses (PRISMA) checklist (17). Main analyses included trigonal-including vs. trigonal-sparing. The subgroup analysis was IDO vs. NDO.

### Search Strategy

We searched relevant RCTs looking at the use of BoNT-A intravesical injection for patients with OAB on PubMed (1997 to Oct. 2020), EMBASE (1997 to Oct. 2020), and the Cochran Central Register for Controlled Trials. Keywords and medical subjects were as follows: “onabotulinumtoxinA (BoNT-A),” “overactive bladder,” “neurogenic detrusor overactivity,” “idiopathic detrusor overactivity,” “randomized controlled trials,” and “meta-analysis.” Two authors completed the whole screening process independently, and if there was any dispute, articles would be sent to another author for evaluation. Relevant reference articles were also included. Furthermore, we reviewed original references of included texts.

### Inclusion Criteria

All of the included RCTs meet the following criteria: (a) the studies should have a connection with the topic “The effect of BoNT-A injections for patients with overactive bladder”; (b) the full useful texts are on RCTs; (c) accurate data could be obtained, and there are similar indicators between the trigone-including group and trigone-sparing group in every RCT. The following studies were excluded: (a) the data were incomplete; (b) the type of study was an abstract, review, comment, case-control, and cohort study. Furthermore, we included the most recently published studies if they described identical experiments. Every study was included if different measures were evaluated.

### Quality Assessment

**Figure 1:**
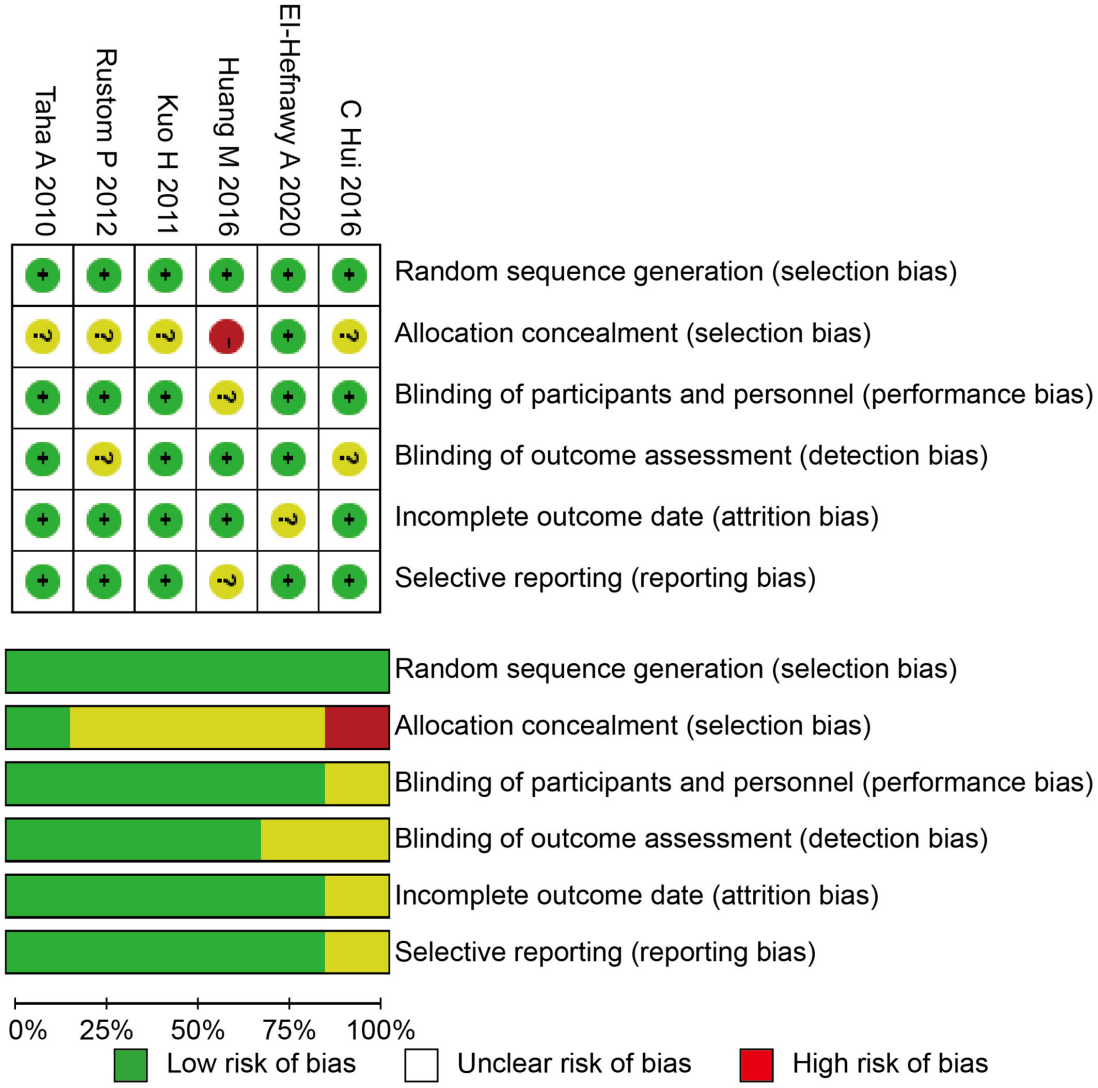
Risk of bias summary and graph. RCT, randomized controlled trials.

We used the Cochrane risk of bias tool to determine the quality of the retrieved RCTs (18). The quality items were selective outcome reporting, blinding, allocation concealment, incomplete outcome data, random sequence generation, and other sources of bias. A graph summarizing the risk of bias was generated based on discussions among the authors (shown in Figure 1). Then, according to the guidelines published in the Cochrane Handbook for Systematic Reviews of Interventions v.5.3.0, the studies were classified qualitatively (19). All of the authors participated in the quality assessment of all RCTs and agreed with the results. Meanwhile, the differences between each RCT were bridged through discussion among authors. All authors participated in the evaluation process and reached a consensus on the final results.

### Data Extraction

The following information was collected for RCTs: (a) the general data in the test; (b) name of the first author; (c) published time; (d) the design of study and size of the sample; (e) the efficacious data in every article that changes in the following parameters, such as the impact on patient symptom score; impact on complete dryness rate; impact on change in the number of incontinence episodes; impact on detrusor pressure at maximum flow rate; impact on volume at the first desire to void; impact in maximum cystometric capacity; impact on post-void residual volume. Finally, another author checked the data extracted from the text. Meanwhile, our team cross-checked references and data for each included study to ensure there were no overlapping data and to maintain the meta-analysis integrity.

### Statistical Analysis and Meta-Analysis

The abstracted data were analyzed with Review Manager 5.1.0 (The Cochrane Collaboration, London, UK) (19). The mean difference (MD) with a 95% confidence interval (CI) was utilized to analyze the continuous data and the odds ratio (OR), and 95% CI was applied to analyze the dichotomous data among the different groups. The chi-square based on *Q* statistic was performed to check the heterogeneity among the studies, and the result was recognized as significant at *P* < 0.05. The fixed-effects model was used and considered to be homogeneous if the result was *p*-value > 0.05. We utilized the *I*^2^ statistic to analyze inconsistent results, and it can reflect the proportion of heterogeneity across trials. In this meta-analysis, it is not necessary to have ethical approval and patient consent because all the data were acquired from articles that have already been published. When the *I*^2^ < 50%, this indicated that there was no significant heterogeneity and the fixed-effects model (Mantel-Haenszel method) would be used. And we performed the random-effects model (DerSimonian and Laird method) when the heterogeneity of the data could not be explained (*p* < 0.05, *I*^2^ > 50%).

## Results

### Characteristics of the Individual Studies

**Figure 2:**
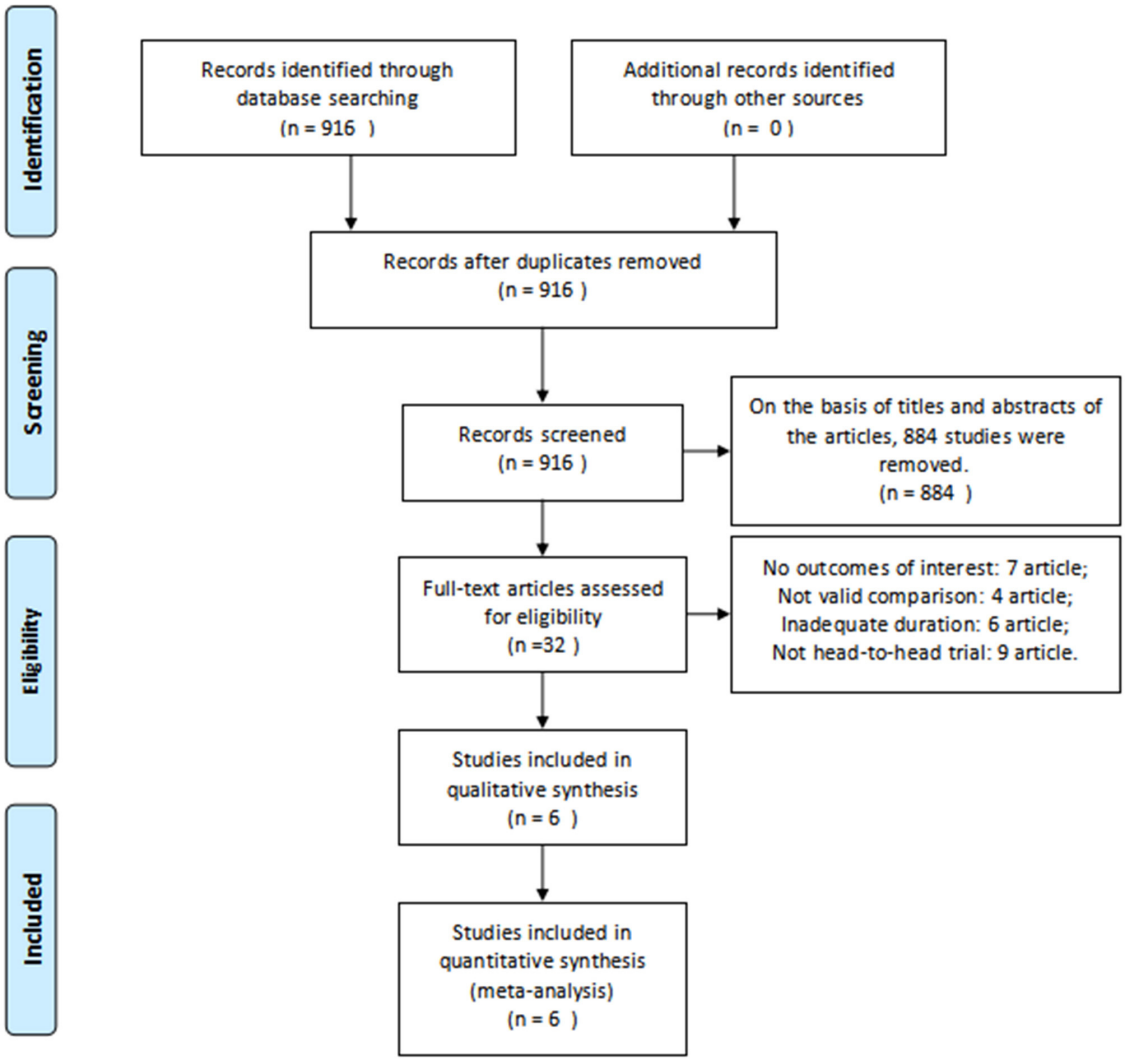
Flowchart of the study selection process. RCT, randomized controlled trials.

Based on the inclusion criteria above, we found 916 articles in the database. After reviewing the abstracts, 578 duplicate articles and 306 studies were removed after reviewing the titles and abstracts of the articles. In total, 26 studies were ruled out for a lack of useful data. Finally, six RCTs (20–25) involving 437 patients were included in our analysis. A detailed flowchart showing the selection process is shown in Figure 2. Table 1 shows the baseline characteristics of studies.

**Table 1:** Study and patient characteristics.

**References**	**Country**	**Experimental group**	**Control group**	**Sample size**		**The doses of therapy (weeks)**		**Inclusion criteria**	**Exclusion criteria**
				**Experimental group (male/female)**	**Control group (male/female)**			
Hui et al. (21)	China	Include trigone	Exclude trigone	47 (28/19)	44 (23/21)	160 U detrusor + 40 U trigone	200 U detrusor	At least 18 years old with various neurogenic disorders; urodynamic DO with urinary incontinence; an inadequate response or intolerance to oral anticholinergic drugs; participants or their caregiver could perform clean intermittent catheterization.	An allergy to BoNT-A; women were pregnant, lactating or planning to become pregnant during the course of the trial; acute urinary tract infection.
El-Hefnawy et al. (20)	Egypt	Include trigone	Exclude trigone	51 (9/42)	52 (12/40)	100 U at 20 sites onto detrusor and trigone	100 U at 20 sites onto detrusor	All patients had been refractory to treatment with antimuscarinics for 2 months.	Age <18 years old; neurogenic DO; evidence of obstructed urinary flow in absence of prolapse; mixed urinary incontinence; associated urethral pathology; associated bladder pathology; active UTI as evidenced by positive urine culture; and previous intravesical Botox injection.
Huang et al. (22)	China	Include trigone	Exclude trigone	41 (17/24)	39 (13/26)	160 U detrusor + 40 U trigone	200 U detrusor	Presence of DO and DESD; and inadequate response or intolerance to oral anti-muscarinic agent or spasmolytic agents, skeletal muscle relaxant and alpha blockers.	Allergy to BoNT-A; coagulopathy disease and myasthenia gravis; acute urinary tract infection; other causes of bladder outlet obstruction; and previous sphincterotomy.
Kuo (23)	China	Include trigone	Exclude trigone	68 (31/37)	37 (17/20)	75 U detrusor + 25 U trigone	100 U detrusor	Aged 18 years or more, with urodynamic DO and at least one episode of urgency or UUI per day as recorded in the 7-day voiding diary.	Neurogenic bladder, urodynamic ally confirmed bladder outlet obstruction, prior pelvic surgery, anti-incontinence surgery, or urinary tract infection.
Manecksha et al. (24)	Ireland	Include trigone	Exclude trigone	11 (1/10)	11 (2/9)	25 U at 15 sites onto detrusor and 5 sites onto trigone	25 U at 20 sites onto detrusor	Aged ≥17 yr with urodynamic-confirmed detrusor overactivity, who had failed ≥6 wk anticholinergic therapy or discontinued therapy due to intolerability	Infection and pregnancy; Patients previously injected with BoNT-A; Patients with any neurologic condition or coagulopathies; as were men with clinical or urodynamic evidence of bladder outflow obstruction.
Abdel-Meguid (25)	Egypt	Include trigone	Exclude trigone	18 (17/1)	18 (17/1)	200 U detrusor + 100 U trigone	300 U detrusor	Adults with SCI, neurogenic urinary incontinence and NDO refractory to anticholinergic medications	Patients who refused CIC or refused to discontinue anticholinergics, those who received previous BoNT-A bladder injections and those with uncontrolled urinary tract infection

*DO, detrusor overactivity; BoNT-A, botulinum toxin A; UTI, urinary tract infection; DESD, detrusor external sphincter dyssynergia; SCI, spinal cord injury; NDO, neurogenic detrusor overactivity; CIC, clean intermittent catheterization*.

### Quality of the Individual Studies

**Figure 3:**
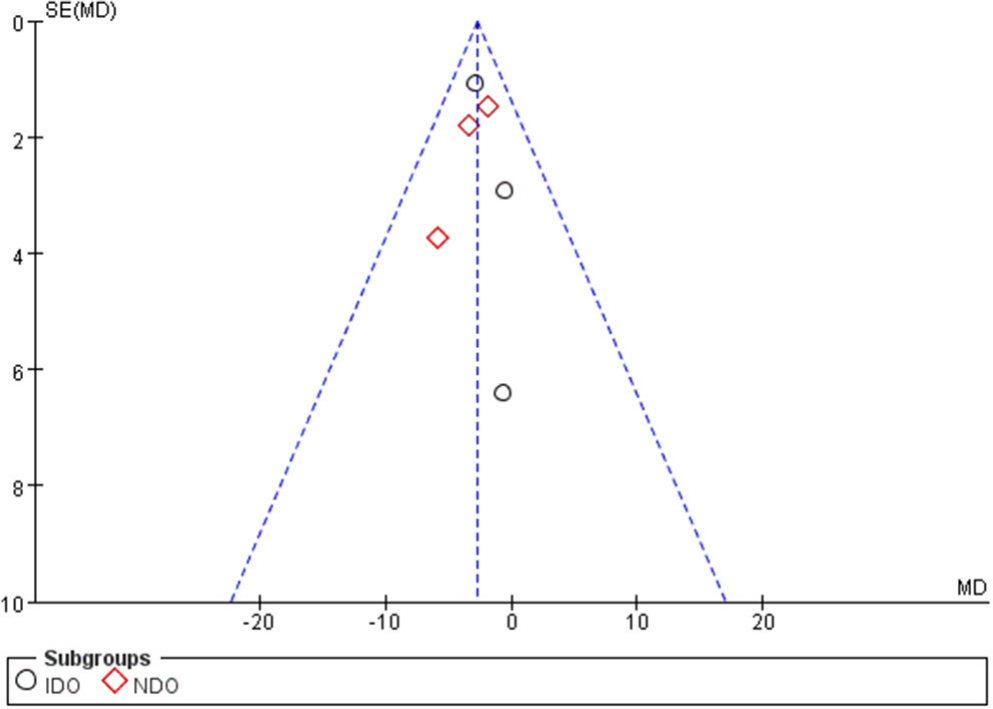
Funnel plot of the studies included in our meta-analysis. MD, mean difference; SE, standard error.

All of the included studies in the systematic review and meta-analysis were high-quality RCTs. Figure 1 presents a graphical summary of the risk bias. We have found that their randomization process has been developed in all studies. All RCTs were effective and determined the best sample size. The funnel plot displayed the conclusion of a qualitative estimation of publication bias of each RCT (Figure 3). Table 2 showed the specific inclusion and exclusion criteria.

**Table 2:** Criteria for considering studies for the review based on the population, intervention, comparator, outcomes, and study designs (PICOS) structure.

	**Population**	**Intervention**	**Comparator**	**Outcomes**	**Study designs**
Inclusion criteria	Aged ≥ 17 years oldwith SCI, neurogenic urinary incontinence and NDO refractory, an inadequate response or intolerance to oral anticholinergic drugs and so on.	BoNT-A into the detrusor and the trigone.	BoNT-A into the detrusor, excluding the trigone.	Impact on patient symptom score; impact on complete dryness rate; impact on change in number of incontinence episodes; impact on detrusor pressure at maximum flow rate; impact on volume at the first desire to void; impact in maximum cystometric capacity; impact on post-viod residual volume.	RCT
Exclusion criteria	Age <17 years old; an allergy to BoNT-A; Infection and pregnancy; mixed urinary incontinence; other causes of bladder outlet obstruction; previous intravesical Botox injection and so on.	Other therapy.	Other therapy.	Qualitative outcomes such as inadequate indicators and others;	Observational study, letters, comments, reviews, and animal experiment.

*BoNT-A, onabotulinumtoxinA; RCT, randomized controlled trial; SCI, spinal cord injury; NDO, neurogenic detrusor overactivity*.

#### Effectiveness

We analyzed the differences in the mean score of each domain for OAB to identify the efficacy of treatment with BoNT-A. Also, we analyzed the differences in treatment effects of BoNT-A for NDO and IDO.

##### Impact on Patient Symptom Score

**Figure 4:**
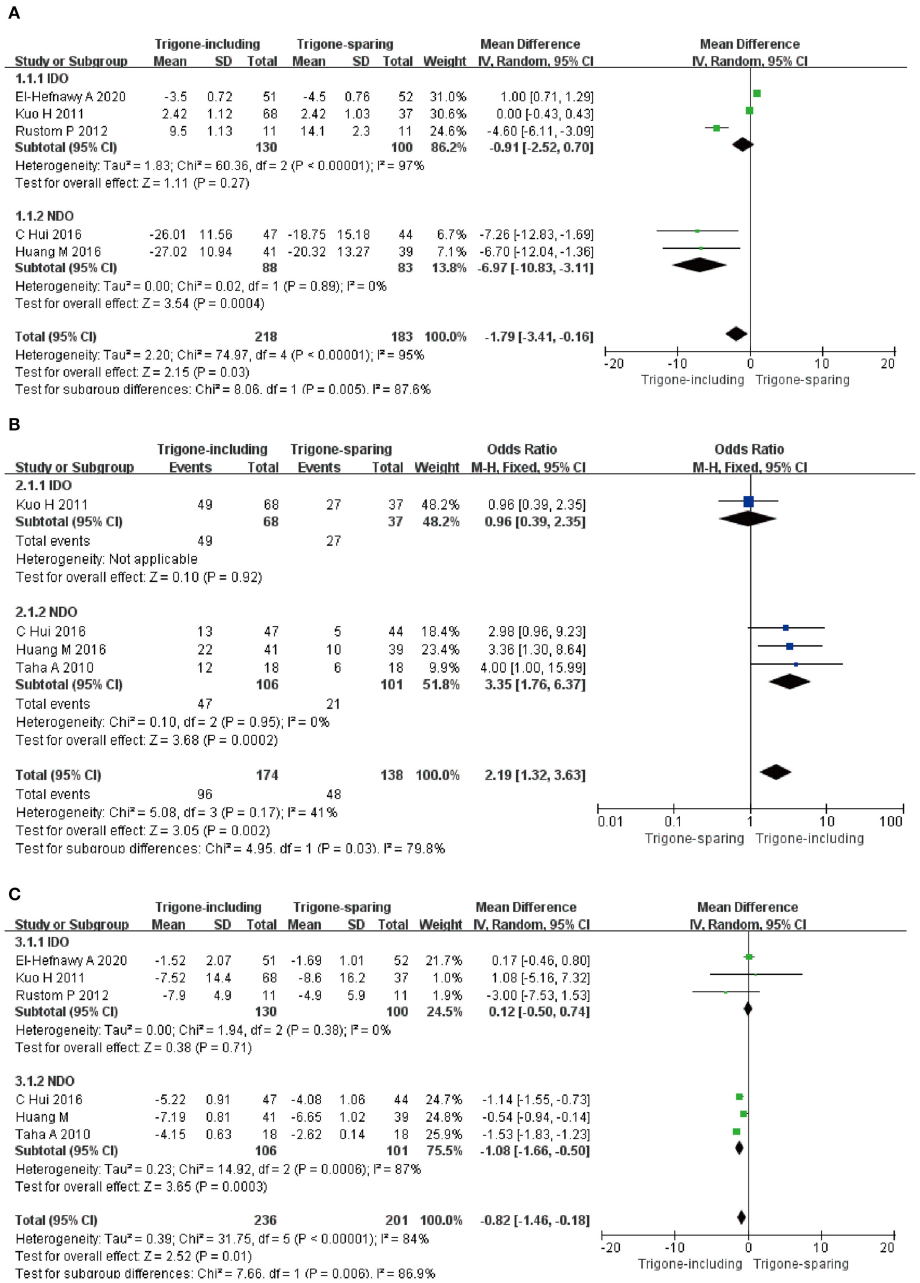
Forest plots showing changes between two groups in the impact on **(A)** patient symptom score, **(B)** complete dryness rate, and **(C)** change in the number of incontinence episodes; NDO, neurogenic detrusor overactivity; IDO, idiopathic detrusor overactivity; SD, standard deviation; IV, inverse variance; CI, confidence interval; df, degrees of freedom.

Five RCTs involving 401 patients (218 in the trigone-including group and 183 in the trigone-sparing group) recorded the changes in impact on patient symptom score (Figure 4A). Since *P* < 0.05, we employed a random-effects model, which reflected an MD of −1.79 (95 CI%: −3.41 to −0.16, *P* = 0.03). The results suggest that the trigone-including group showed statistical differences in the impact on patient symptom score compared with the trigone-sparing group for OAB. A subgroup analysis revealed that trigone-including intradetrusor injection had no marked difference in the impact on patient symptom score compared with the trigone-sparing group for IDO (MD = −0.91, 95% CI: −2.28 to −0.70, *P* = 0.27). For NDO, it showed a significant difference between the two groups in the change of the impact on patient symptom score (MD = −6.97, 95% CI: −10.83 to −3.11, *P* = 0.0004).

##### Impact on Complete Dryness Rate

Four RCTs involving 312 patients (174 in trigone-including group and 138 in trigone-sparing group) recorded the change in impact on complete dryness rate (Figure 4B). Since *P* > 0.05, we employed a fixed-effects model, which reflected an MD of 2.19 (95% CI: 1.32 to 3.63, *P* = 0.002). The results suggest that the trigone-including group showed statistical differences in the impact on complete dryness rate compared to the trigone-sparing group for OAB. A subgroup analysis revealed that trigone-including intradetrusor injection showed a significantly different impact on patient symptom score compared with the trigone-sparing group for NDO (MD = 3.35, 95% CI: 1.76 to 6.37, *P* = 0.0002).

##### Impact on Change in Number of Incontinence Episodes

A total of six RCTs involving 437 patients (236 in the trigone-including group and 201 in the trigone-sparing group) recorded the changes in impact on change in the number of incontinence episodes (Figure 4C). A random-effects model showed an MD of −0.82 (95% CI: −1.46 to 0.18, *P* = 0.01). It proved that the trigone-including group showed statistical differences in terms of the impact on change in the number of incontinence episodes for OAB. For IDO, a subgroup analysis revealed that trigone-including intradetrusor injection showed no differences in the impact on patient symptom score compared with the trigone-sparing group for IDO (MD = 0.12, 95% CI: −0.50 to 0.74, *P* = 0.71). For NDO, it showed statistical differences between the two groups (MD = −1.08, 95% CI: −1.66 to −0.50, *P* = 0.0003).

##### Impact on Detrusor Pressure at the Maximum Flow Rate

**Figure 5:**
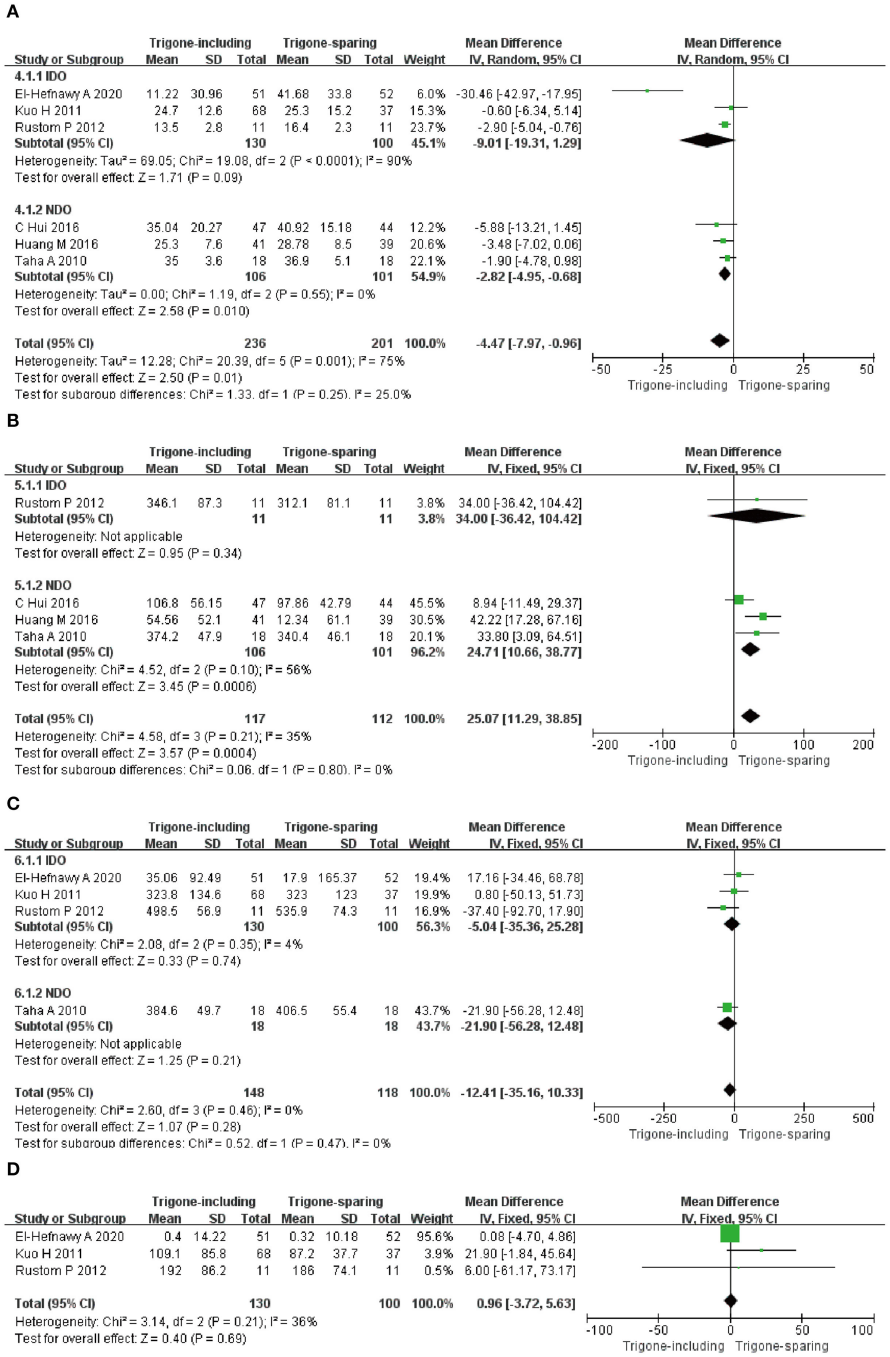
Forest plots showing changes between two groups in **(A)** impact on detrusor pressure at maximum flow rate, **(B)** impact on volume at the first desire to void, **(C)** impact in maximum cystometric capacity, **(D)** impact on post-void residual volume; NDO, neurogenic detrusor overactivity; IDO, idiopathic detrusor overactivity; SD, standard deviation; IV, inverse variance; CI, confidence interval; df, degrees of freedom.

A total of six RCTs involving 437 patients (236 in the trigone-including group and 201 in the trigone-sparing group) recorded the changes in impact on detrusor pressure at the maximum flow rate (Figure 5A). Since *P* < 0.05, we employed a random-effects model, which reflected an MD of −4.47 (95% CI: −7.97 to −0.96, *P* = 0.01). The results suggest that the trigone-including group showed statistical differences in the impact on detrusor pressure at maximum flow rate compared with the trigone-sparing group for OAB. A subgroup analysis revealed that a trigone-including intradetrusor injection showed no marked differences in the impact on detrusor pressure at the maximum flow rate compared with the trigone-sparing group for IDO (MD = −9.01, 95% CI: −19.31 to 1.29, *P* = 0.09). Also, there were statistical differences in terms of impact on detrusor pressure at the maximum flow rate between the two groups for NDO (MD = −2.82, 95% CI: −4.95 to −0.68, *P* = 0.01).

##### The Changes in Differences in the Impact on Volume at the First Desire to Void

A total of four RCTs involving 318 patients (117 in the trigone-including group and 201 in the trigone-sparing group) recorded the changes in impact on *volume at the first desire to void* (Figure 5B). Since *P* > 0.05, we employed a fixed-effects model, which reflected an MD of 25.07 (95% CI: 11.29 to 38.85, *P* = 0.0004). The results suggest that the trigone-including group showed statistical differences in the impact on the volume of the first desire to void compared with the trigone-sparing group for OAB. For NDO, a subgroup analysis showed statistical differences between the two groups (MD = 24.71, 95% CI: 10.66 to 38.77, *P* = 0.0006).

##### Impact in Maximum Cystometric Capacity

A total of four RCTs involving 266 patients (148 in trigone-including group and 118 in trigone-sparing group) recorded the changes in impact in terms of maximum cystometric capacity (Figure 5C). A fixed-effects model showed an MD of −12.41, 95% CI: −35.16 to 10.33, *P* = 0.28. It proved that the trigone-including group showed no differences in terms of the change in impact in maximum cystometric capacity. A fixed-effects model also showed that the trigone-including group showed no marked differences in the impact in maximum cystometric capacity with the trigone-sparing group for IDO (MD = −5.04, 95% CI: −35.36 to 25.28, *P* = 0.74).

##### Impact on Post-void Residual Volume

A total of three RCTs involving 230 participants (130 in the trigone-including group and 100 in the trigone-sparing group) recorded the changes in impact on the post-void residual volume (Figure 5D). A fixed-effects model showed an MD of 0.96, 95% CI: −3.72 to −5.63, *P* = 0.69. There were no statistical differences in terms of impact on post-void residual volume between the two groups.

### Safety

#### Hematuria; General Weakness; Bladder Discomfort; Incidence of Large Post-void Residual; Urinary Tract Infection; Difficulty of Voiding

**Figure 6:**
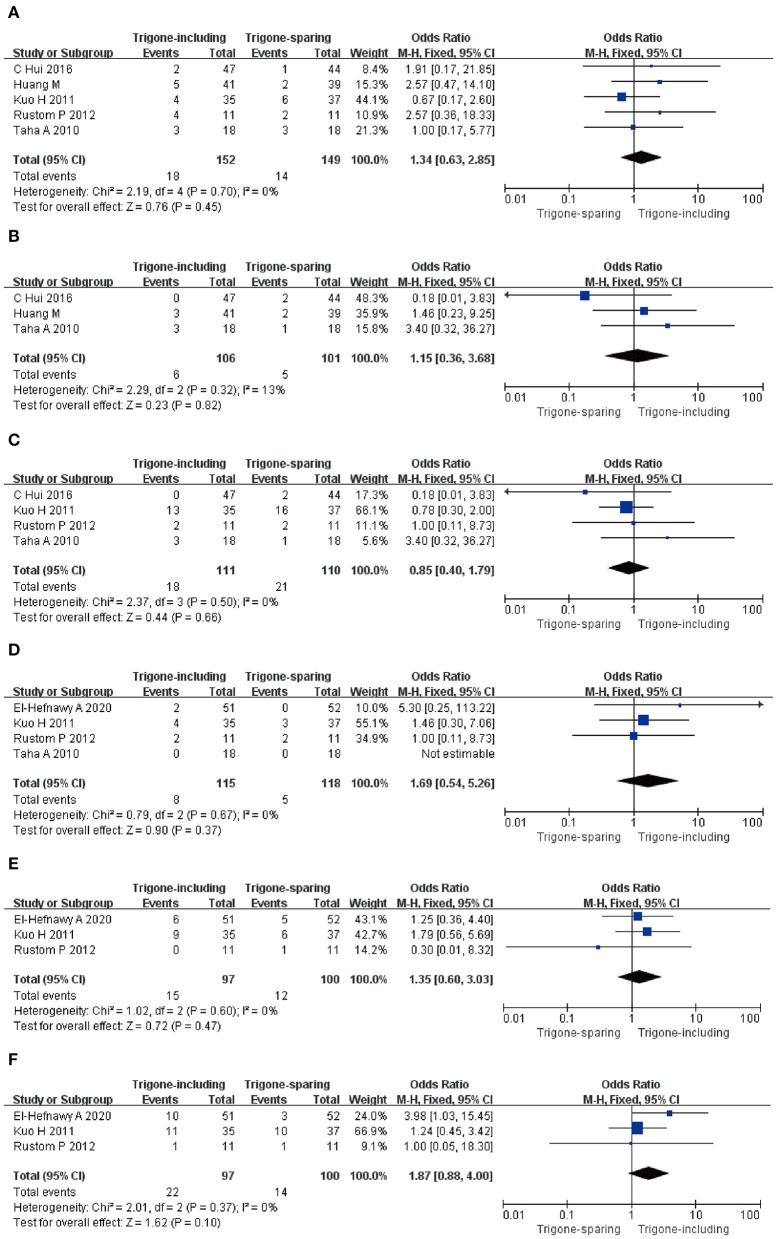
Forest plots showing changes between two groups in **(A)** hematuria, **(B)** general weakness, **(C)** bladder discomfort, **(D)** incidence of large post-void residual, **(E)** urinary tract infection, and **(F)** difficulty of voiding; SD, standard deviation; IV, inverse variance; CI, confidence interval; df, degrees of freedom.

A total of five RCTs, including 301 participants (152 in the trigone-including group and 149 in the trigone-sparing group) were involved in the research for Hematuria. The OR of the study was 1.34, and the 95% CI was 0.63–2.85 (*P* = 0.45); three RCTs analyzed the incidence of the general weakness of 207 patients (106 in the trigone-including group and 101 in the trigone-sparing group) (OR = 1.15; 95%CI = 0.36–3.68; *P* = 0.82); four RCTs including 221 participants (111 in the trigone-including group and 110 in trigone-sparing group) were involved in the research for bladder discomfort (OR = 0.85; 95%CI = 0.40–1.79; *P* = 0.66); four RCTs including 233 patients (115 in the trigone-including group and 118 in the trigone-sparing group) were involved in the research for the incidence of large post-void residual (OR = 1.69; 95%CI = 0.54–5.26; *P* = 0.37); three RCTs analyzed the incidence of urinary tract infection of 197 patients (97 in the trigone-including group and 100 in the trigone-sparing group) (OR = 1.35, 95%CI = 0.60–3.03 *P* = 0.47); and three RCTs including 197 participants (97 in the trigone-including group and 100 in the trigone-sparing group) were involved in the research for difficulty of voiding (OR = 1.87, 95%CI = 0.88–4.00 *P* =0.10). These results indicate that there is no significant difference between the two groups in terms of Hematuria; general weakness; bladder discomfort; incidence of large post-void residual; urinary tract infection; difficulty of voiding. There were no statistical differences in terms of side-effects between the two groups (Figure 6).

## Discussion

OAB is a series of clinical symptoms by urgency frequency with or without UUI that are caused by neurogenic or non-neurogenic factors (4). Behavioral and bladder training, drug therapy, botulinum toxin, electrical stimulation, biofeedback, or surgery can be used to treat patients with OAB. OAB is an illness that greatly compromises the quality of life of patients. Due to less effectiveness and poor adherence to first and second-line treatments, the main objective is the search for new treatments in OAB therapy. Nowadays, literature research reveals that BoNT-A intravesical injection was recommended as a third-line treatment of OAB (26, 27). Intravesical BoNT-A injections may represent a treatment modality for patients with NDO and IDO. Local drug delivery is one of the efficacious ways to lessen the side effects of systemic administration compared with anticholinergic therapy (inadequate efficacy or intolerable side effects).

In summary, for OAB, the trigone-including group showed differences in the patient symptom score (*p* = 0.03), complete dryness rate (*p* = 0.002), frequency of incontinence episodes (*p* = 0.01), detrusor pressure at maximum flow rate (*p* = 0.01), and volume at the first desire to void (*p* = 0.0004) compared with the trigone-sparing group. A trigone-including injection can improve the patient symptom score. Meanwhile, the higher complete dryness rate and a lower frequency of incontinence episodes in patients with a trigone-including intradetrusor injection can also increase the quality of life of patients. At the same time, the analysis demonstrated that the trigone-including group showed a similar result to the trigone-sparing group in terms of the aspects of maximum cystometric capacity (*p* = 0.28) and post-void residual volume (*p* = 0.69).

The dose of BoNT-A can affect the therapeutic effect. In this study, the high dose (150–300 U) of BoNT-A was used for the treatment of NDO, and it showed marked differences between the trigone-including group and trigone-sparing group. At the same time, there were no differences between the groups for treatment of IDO when a low dose (25–150 U) of BoNT-A was used. The dose of BoNT-A was different for the treatment of NDO and IDO, and we cannot be sure which one dose of BoNT-A was suitable for the treatment of OAB. The dose of 50 U demonstrated consistently lower non-sustained efficacy than doses of 100 U or higher (28). Meanwhile, the 150 U does or higher had the same clinical curative effect and not provided additional effect. Previously research can also show the high doses of BoNT-A can increase the risk of post-void residual(PVR) and associated urinary tract infection(UTI) in patients with OAB (29). Thus, we recommend to use a dose of 100 U for treatment of unexplained OAB.

In subgroup analyses, a trigone-including intradetrusor injection demonstrated significant improvement in patient symptom score (*p* = 0.0004), complete dryness rate (*p* = 0.0002), frequency of incontinence episodes (*p* = 0.0003), detrusor pressure at maximum flow rate (*p* = 0.01), and volume at the first desire to void (*p* = 0.0006) compared with the trigone-sparing group for treatment of NDO. That, the two methods did not differ for the treatment of IDO. This result showed that the trigone is potentially the optimal region where therapy should be directed.

BoNT-A, a potent neurotoxic protein, is a kind of neurotoxin that comes from the aerobic bacterium botulinum. There are several types of botulinum toxin, and type A is the most effective regarding duration. There are two influencing mechanisms: (a) it can regulate acetylcholine release from presynaptic neurons and inhibit the contractions of the detrusor or urethral sphincter; (b) it also regulates sensory nerve function by blocking the release of various noxious neurotransmitters, including adenosine triphosphate, calcitonin gene-related peptide, calcitonin gene-related peptide, and substance P (14). The BoNT-A consists of two pieces: 50-kDa light chain and a 100-kDa heavy endocytosis (30). The light chain of BoNT-A has biological activities. It cleaves the synaptosome-associated protein in the presynaptic nerve terminal and inhibits the release of acetylcholine by disrupting the fusion of vesicles with the neuron's cell membrane. This could finally lead to flaccid paralysis of muscles (31, 32). BoNT-A was injected into multiple sites within the bladder wall directly by cystoscopy. The most common adverse reactions following the administration of the toxin are urinary retention and urinary tract infection.

NDO was considered in relation to neural dysfunction. The motor neurons of the bladder include: (a) the sympathetic nerves, which are associated with an increment of bladder outlet obstruction during the storage phase of the micturition cycle; and (b) the parasympathetic nerves, which can produce spontaneous action potential and initiating spontaneous contraction of the bladder. New research indicates that the trigone was most densely innervated in the bladder. These nerves respond to passive distension and active contraction of the bladder and are the sensory component during a normal micturition cycle (33). Botulinum toxins have a direct role in the sensory nerve endings as well as the synaptic nerve junctions. Intravesical injections of BTX-A are useful for the treatment of bladder overactivity without the systemic adverse effects associated with pharmacotherapy (34). Thus, using trigonal injection might play a central role in treating NDO.

This meta-analysis found that the inclusion of the trigone in the injection pattern improves the outcome. Moreover, other studies reported that pressure directly on the trigone results in sensations of urgency, and trigonal injection of lidocaine or surgical denervation of the trigone relieved urgency in patients with OAB (35). This makes sense, as it is known that there is a rich source of afferent innervation in the trigone. These afferents originate primarily *via* the hypogastric nerve, suggesting a sympathetic nerve origin for the afferents responsible for urgency sensation arising from the trigone, whereas filling sensations arising from the bladder body are likely mixed in origin (parasympathetic and sympathetic) (36). Also, this meta-analysis revealed a greater effect in NDO than IDO, where it well-known that sensory nerve function is altered. We know that the sensory nerves innervate the bladder and are tuned to be mechanosensitive and that this signal activates areas of the spinal cord responsible for relaying sensation (37). Furthermore, studies have shown that the vortex infused into the bladder lumen is able to significantly attenuate this mechanosensitive bladder nerve activity without affecting the pressure/volume relationship during bladder filling (38). Thus, the analysis and conclusions of this meta-analysis are consistent with previous anatomical and physiological findings.

The incidence of adverse events between the trigone-including group and trigone-sparing group included hematuria (*p* = 0.45) and general weakness (*p* = 0.82); bladder discomfort (*p* = 0.66); incidence of large post-void residual (*p* = 0.37); urinary tract infection (*p* = 0.47); and difficulty of voiding (*p* = 0.10). The adverse events rates were similar in both groups. It is worth mentioning that trigone-including injections of BoNT-A may cause vesicoureteral reflux in theory; however, Mascarenhas et al. showed it did not cause vesicoureteral reflux in patients with OAB (39, 40). Meanwhile, the single injection of BTX-A only inflicts minor damage and does not induce bladder fibrosis or cause bladder tissue damage. The adverse reactions from long-term treatment remain to be elucidated (41).

One final but important point is that the results of injecting BoNT-A for treatment of OAB are affected by the dose of BoNT-A and the depth of injection. The work of Jo et al. presents a subgroup analysis according to the dose of BoNT-A. When a dosage of 200–300 units of BoNT-A was used, the endpoints of the symptom score, complete dryness, and frequency of incontinence episodes improved significantly in the trigone-including group. The depth of injection was determined by the surgeon with flexible or rigid cystoscopy. Jo et al. also reported that the depth of injection had no significant impact on the efficacy and safety between submucosal and detrusor injection (42). Submucosal injections have some advantages. It is easy to operate, does not require deep injection of the needle, and reduces the damage of the blood vessel. Thus, we recommend submucosal injection as a standard approach for the treatment of OAB (43).

This meta-analysis included six RCTs and concentrating on the efficacy and safety of BoNT-A for the treatment of overactive bladder. Compared with previous studies, our study had some advantages; the data were derived from randomized, double-blind controlled trials. Meanwhile, we also separately analyze the differences between IDO and NDO. However, this study also has some limitations, which reflect the common limitations of other systematic reviews and meta-analyses. This article did not include numerous patients and RCTs, which may affect our meta-analysis quality. Meanwhile, we will need more appropriate high-quality trials to improve the accuracy of results.

## Conclusions

In summary, our meta-analysis has demonstrated that trigone-including BoNT-A injection was more effective compared with the trigone-sparing injection for the treatment of OAB, especially for NDO. There had been similar safety between the two methods.

## Data Availability Statement

The original contributions presented in the study are included in the article/supplementary material, further inquiries can be directed to the corresponding author/s.

## Author Contributions

TC and TD: conceptualization and methodology. ZZ: data curation. TC and YW: writing—original draft preparation. YL: visualization. ZG: investigation. XZ: supervision. YZ: software. JW and LD: validation. YC: writing—reviewing and editing. All authors contributed to the article and approved the submitted version.

## Funding

This work was supported by the National Nature Science Foundation of China (Nos. 81870525; 81801429; and 81572835), Taishan Scholars Program of Shandong Province (No. tsqn201909199), Beijing Municipal Administration of Hospitals' Ascent Plan (Code: DFL20190502), and Beijing Municipal Administration of Hospitals Clinical Medicine Development of Special Funding Support (Code: ZYLX201820).

## Conflict of Interest

The authors declare that the research was conducted in the absence of any commercial or financial relationships that could be construed as a potential conflict of interest.

## Publisher's Note

All claims expressed in this article are solely those of the authors and do not necessarily represent those of their affiliated organizations, or those of the publisher, the editors and the reviewers. Any product that may be evaluated in this article, or claim that may be made by its manufacturer, is not guaranteed or endorsed by the publisher.

## Abbreviations

OAB: overactive bladder
UUI: urgency urinary incontinence
UTI: urinary tract infection
NDO: neurogenic detrusor overactivity
DO: overactivity
SCI: spinal cord injury
BoNT-A: onabotulinumtoxinA
IDO: idiopathic detrusor overactivity
RCT: randomized controlled trial
MD: mean difference
SE: standard error
SD: standard deviation
IV: inverse variance
CI: confidence interval
df: degrees of freedom
OR: odds ratio.
